# Identification of Genes Associated with Smad3-dependent Renal Injury by RNA-seq-based Transcriptome Analysis

**DOI:** 10.1038/srep17901

**Published:** 2015-12-09

**Authors:** Qin Zhou, Yuanyan Xiong, Xiao R. Huang, Patrick Tang, Xueqing Yu, Hui Y. Lan

**Affiliations:** 1 Department of Nephrology, The First Affiliated Hospital, Sun Yat-sen University, Guangzhou, China; 2 Li Ka Shing Institute of Health Sciences and Department of Medicine & Therapeutics, the Chinese University of Hong Kong, Hong Kong, China; 3 Shenzhen Research Institute, the Chinese University of Hong Kong, Shenzhen, China; 4 State Key Laboratory for Biocontrol, Sun Yat-sen University, Guangzhou, China; 5 SYSU-CMU Shunde International Joint Research Institute, Guangzhou, China

## Abstract

Transforming growth factor-β/Smad3 signaling plays a critical role in the process of chronic kidney disease (CKD), but targeting Smad3 systematically may cause autoimmune disease by impairing immunity. In this study, we used whole-transcriptome RNA-sequencing to identify the differential gene expression profile, gene ontology, pathways, and alternative splicing related to TGF-β/Smad3 in CKD. To explore common dysregulation of genes associated with Smad3-depednent renal injury, kidney tissues of Smad3 wild-type and knockout mice with immune (anti-glomerular basement membrane glomerulonephritis) and non-immune (obstructive nephropathy)-mediated CKD were used for RNA-sequencing analysis. Totally 1922 differentially expressed genes (DEGs) were commonly found in these CKD models. The up-regulated genes are inflammatory and immune response associated, while decreased genes are material or electron transportation and metabolism related. Only 9 common DEGs were found to be Smad3-dependent in two models, including 6 immunoglobulin genes (Ighg1, Ighg2c, Igkv12-41, Ighv14-3, Ighv5-6 and Ighg2b) and 3 metabolic genes (Ugt2b37, Slc22a19, and Mfsd2a). Our results identify transcriptomes associated with renal injury may represent a common mechanism for the pathogenesis of CKD and reveal novel Smad3 associated transcriptomes in the development of CKD.

Chronic Kidney Disease (CKD) becomes a major problem and healthcare burden in China, which affects 10.8% of the population with high prevalence and low awareness according to a national survey in 2012^1^,^2^. Renal inflammation and fibrosis are the common manifestations of CKD. Transforming growth factor-β1 (TGF-β1), a profibrogenic cytokine, has an important biological role in the induction of organ inflammation and fibrosis by activating the downstream Smad proteins, especially Smad3^3^,^4^,^5^,^6^. Although Smad3 is a critical transcription factor which conveys signals from TGF-β receptors to the nucleus, targeting Smad3 may cause autoimmune disease by impairing immunity^6^,^7^. Thus, alternative approaches to inhibit TGF-β actions should be more precise and can be achieved by identifying the Smad3-dependent transcriptomes in chronic renal injury.

High-throughput RNA sequencing (RNA-Seq) is rapidly emerging as a favorite approach for transcriptome-oriented studies; it permits quantification of genes and products of their processing or partial degradation, and identification of novel coding and non-coding RNA^8^,^9^,^10^. In previous study, we successfully identified long non-coding RNAs in Smad3 wild-type (WT) and knock-out (KO) mouse models of obstructive nephropathy (UUO) and immunologically-induced anti-glomerular basement membrane glomerulonephritis (anti-GBM GN) by using RNA-seq^11^,^12^. Compared to WT mice, a number of transcripts including mRNAs and non-coding RNAs in the diseased kidneys from UUO or anti-GBM GN models were significantly altered in Smad3 KO mice. The present study aimed to compare the profiles of Smad3 related DEGs in these two CKD models

## Results

### RNA-Sequencing of mouse transcriptomes in two CKD models

High-throughput RNA-Seq was used to determine the Smad3-dependent transcriptome profiles of two CKD animal models. Total RNA of kidneys from Smad3 KO and WT mice with UUO or anti-GBM GN were collected for the analysis. As shown in Table 1, after filtering the low quality reads and rRNA sequences from the raw fragments, we obtained over 50 M reads in each sample. Total clean reads were then mapped to mouse genome (mm9) by using Tophat. Approximately, 66.96 ~ 97.62% were successfully mapped to mouse genomic regions by allowing no more than two mismatches in 25 bp segments and only output the alignment results which are no more than at most 10 genomic locations. Among the aligned fragments, 42.65 ~ 87.88% were mapped to unique genomic regions, which were used for further analysis to ensure reliability of analysis results.

### Analyses of differentially expressed genes

The FPKM value was used to quantifying the mRNA expression. We analyze the profiles of DEGs (differentially expressed genes) between disease and normal control groups. Pearson correlation analysis showed that the FPKM values between these groups highly correlated (r = 0.87–0.92, r < 0.01) (Supplementary Fig. S1).

#### Comparison of DEGs related to renal injury in two models

**Figure 1:**
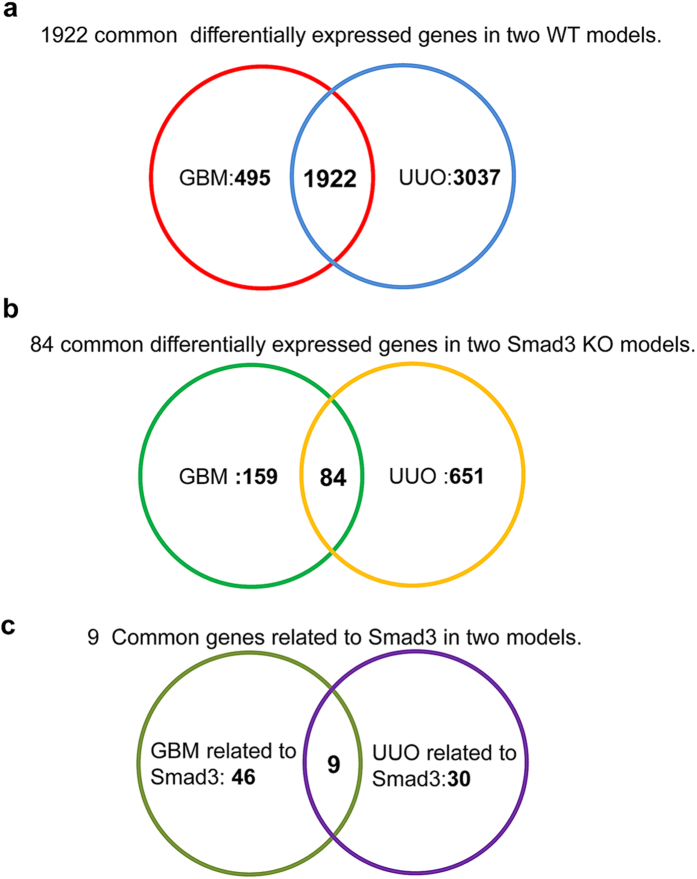
Number of total DEGs in two CKD models of WT and Smad3 KO mice. (**a**) Venn diagram has shown1922 common differentially expressed genes in two WT models. (**b**) Venn diagram of 84 common differentially expressed genes in two Smad3 KO models. (**c**) Venn diagram of 9 common Smad3-related differentially expressed genes in two models. GBM:anti-GBM GN model in wild-type or Smad3 knockout mice; UUO: UUO model in wild-type mice or Smad3 knockout mice.

The analysis of DEGs was shown in Fig. 1a and Table 2, compared to WT-Nor (Smad3 wild-type normal control) group, totally 2417 DEGs were found in WT-GBM (Smad3 wild-type anti-GBM GN model) including 1552 up-regulated and 865 down-regulated DEGs which accounting for 64.21% and 35.79% of the total DEGs. In WT-UUO (Smad3 wild-type UUO model),the up- and down-regulated DEGs were 2898 and 2061,accounting for 58.44% and 41.56% of the total 4959 DEGs detected respectively. These findings indicated that the number of DEGs in UUO is significantly larger than the anti-GBM GN models (4959 vs 2417). In addition, 1922 DEGs were commonly found in the wild-type groups of both models including 1203(62.59%) up-regulated and 719(37.41%) down-regulated genes (all the gene ID and fold changes are listed in Supplementary File S1).

In WT-GBM, Ighg1 (immunoglobulin heavy constant gamma 1, LogFC = 13.29, FDR = 6.93E-30) was largely upregulated when compared to WT-Nor whereas Sftpc (surfactant associated protein C) (LogFC = −11.40, FDR = 5.63E-05) was greatly suppressed. The increase of Ighg1 may be associated with humoral immune response of B cell^13^. Sftpc is an extremely hydrophobic surfactant protein^14^,^15^, deficiency mice of Sftpc leads to inflammation and increased cytokine production^16^. In WT-UUO, FlnA (filamin, alpha, LogFC = 17.25, FDR = 1.40E-54) was the most up-regulated gene compared to the WT-Nor, which encode a widely expressed actin-binding protein that regulates the reorganization of actin cytoskeleton by interacting with integrins, transmembrane receptor complexes, and second messengers^17^, whereas, Sftpc (LogFC = −11.37,FDR = 8.30E-05) was significantly downregulated.

#### Comparison of Smad3-related DEGs in renal injury

Further analysis revealed that, compared with WT mice, many genes were differentially expressed in the kidneys of Smad3 knockout mice during CKD. As shown in Fig. 1b and Table 2, deletion of Smad3 significantly diminished the genes expression diversity in two disease models; the total numbers of DEGs were largely reduced when compared to the WT models. Compared to KO-Nor (normal control of Smad3 knockout mice) group, the total number of DEGs was 243 in KO-GBM (anti-GBM GN model of Smad3 knockout mice) and 735 in KO-UUO (UUO model of Smad3 knockout mice). The up and down-regulated genes were 117 and 126 in KO-GBM, while 400 and 335 were detected in KO-UUO compared to the KO-Nor (Smad3 knockout normal mice). The total common DEGs in two CKD models of Smad3 KO mice were 84, which included 36 up-regulated and 48 down-regulated genes (Table 2). All the comparisons of DEGs in Smad3 KO models were also listed in Supplementary File S1 (including all the gene ID and fold changes).

To identify of Smad3-related genes in two models, DEGs have different expression direction in WT and Smad3 KO mice will be considered as Smad3-related. In these DEGs, 55 genes in anti-GBM GN model and 39 genes in UUO model were Smad3-related and differentially expressed in the WT and Smad3 KO kidneys (Fig. 1c and Table 3). The 9 common Smad3-associated DEGs in two CKD models were including 6 immunoglobulin genes (Ighg1, Ighg2c, Igkv12-41, Ighv14-3, Ighv5-6, and Ighg2b) and 3 metabolic genes (Ugt2b37, Slc22a19, and Mfsd2a).

### Analysis of Gene Ontology and Pathways

**Figure 2:**
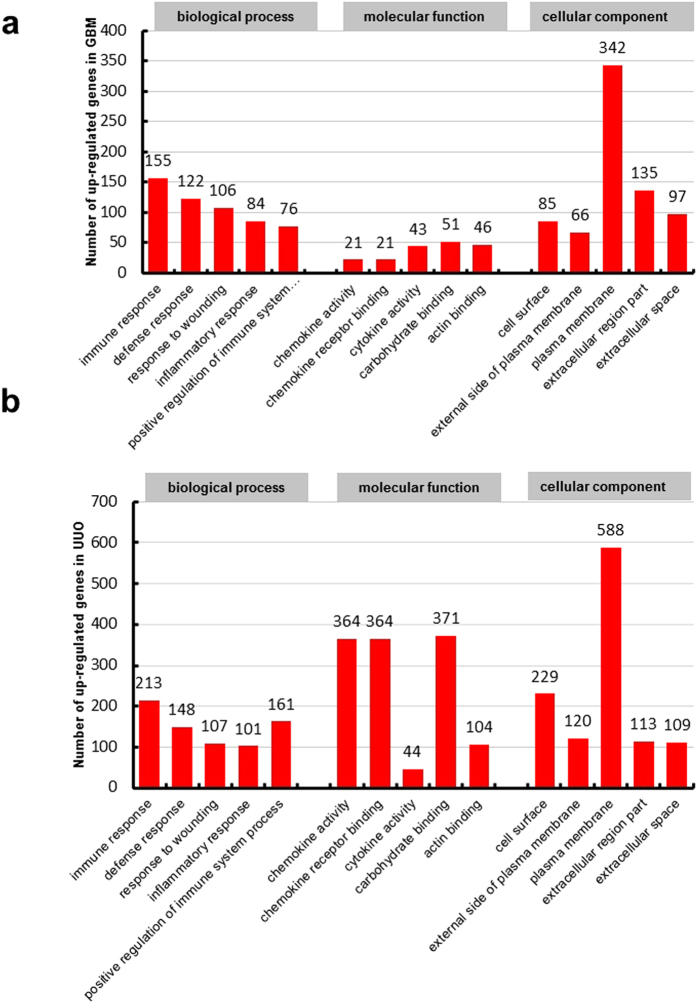
Histogram showing the top five significant GO terms (including biological processes, molecular function and cellular component) of up-regulated genes in WT-GBM (a) or WT-UUO models (b) compared to WT-Nor.WT-UUO:UUO model in wild-type mice; WT-GBM:anti-GBM GN model in wild-type mice; WT-Nor:wild-type normal mice.

The NIH Database for Annotation, Visualization and Integrated Discovery (DAVID) was used to perform gene functional annotation clustering. Gene Ontology describes attributes of genes in three non-overlapping domains of molecular biology which including biological processes, molecular function and cellular component. We summarized the number of significant GO terms that DEGs enriched in Table 4. Because ‘biological process’ is the most widely used subontology of GO to evaluate the functions of gens, we focus on the Go terms of biological process. According to GO terms for biological process in up-regulated genes, we obtained 171 significant GO terms in WT-GBM and 209 in WT-UUO compared to WT-Nor. In two WT models, common up-regulated genes are enriched in 138 GO terms for biological process. The top 5 GO terms of up-regulated genes with lowest FDR in two WT models were shown in Fig. 2a,b, immune response, defense response, response to wounding, inflammatory response, positive regulation of immune system process were the common GO terms of biological processes in two models.

**Figure 3:**
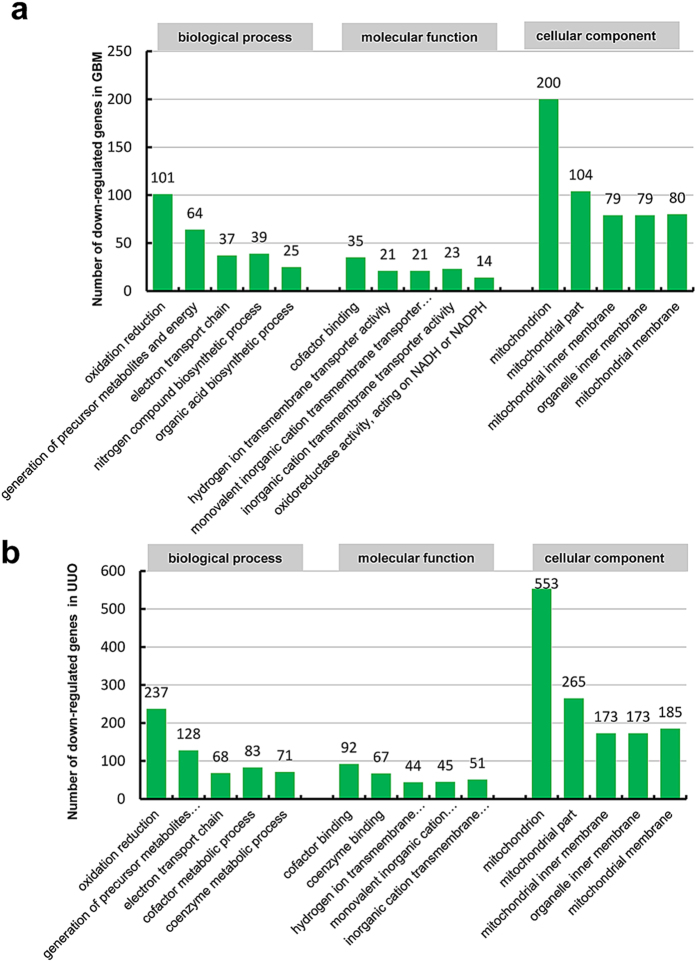
Histogram showing the top five significant GO terms (including biological processes, molecular function and cellular component) of down-regulated genes in WT-GBM (a) or WT-UUO models (b) compared to WT-Nor.WT-UUO:UUO model in wild-type mice; WT-GBM:anti-GBM GN model in wild-type mice; WT-Nor:wild-type normal mice.

According to the GO analysis results of down-regulated genes in Table 4, 26 significant GO terms for biological process were found in WT-GBM and 105 in WT-UUO compared to WT-Nor respectively and the top 5 GO terms of down-regulated genes in two models were shown in Fig. 3a,b, There were 26 common down-regulated genes enriched GO terms in biological process, oxidation reduction, generation of precursor metabolites and energy, electron transport chain are the most significant GO terms of biological processes in two models.

The number of significant GO terms in Smad3 KO models is much less than WT models. Only a few significant GO terms were found in these two KO models, that may be due to less DEGs were found in KO models. For up-regulated genes, we find only one common significant GO term of cellular component (extracellular region part) in KO-UUO and KO-GBM. For down-regulated genes, one biological process (acute inflammatory response), one cellular component (extracellular space) and 4 molecular function (endopeptidase inhibitor activity, peptidase inhibitor activity, enzyme inhibitor activity, serine-type endopeptidase inhibitor activity) were common significant GO terms in KO-UUO and KO-GBM. Additional details for GO analysis of DEGs in WT and Smad3 knockout models were listed in Supplementary File S2 and S3 (including all the GO terms and gene ID).

**Figure 4:**
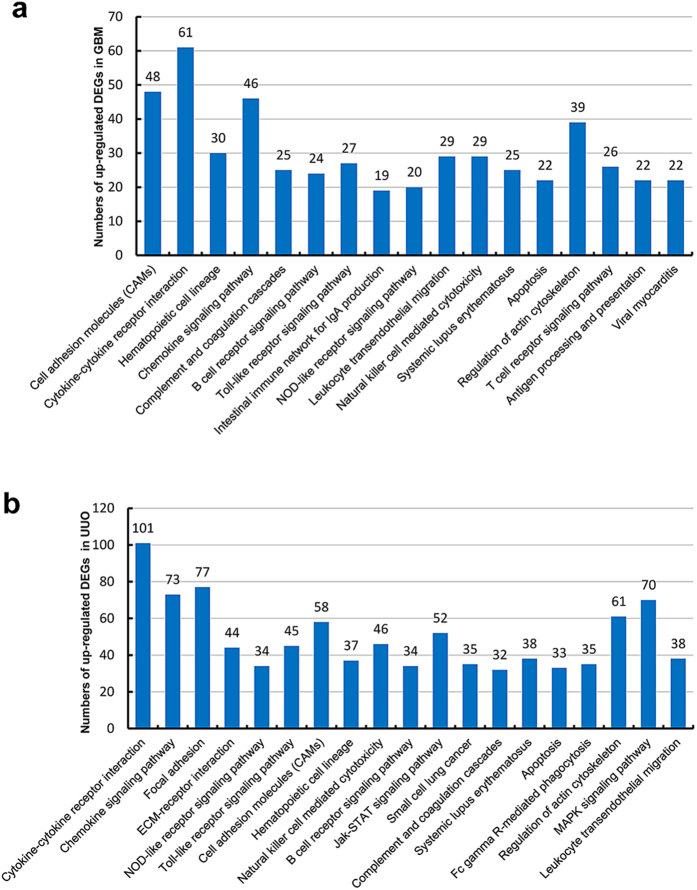
Histogram showing all the significant KEGG pathways of up-regulated genes in WT-GBM (a) or WT-UUO models (b) compared to WT-Nor.WT-UUO:UUO model in wild-type mice;WT-GBM:anti-GBM GN model in wild-type mice; WT-Nor:wild-type normal mice.

**Figure 5:**
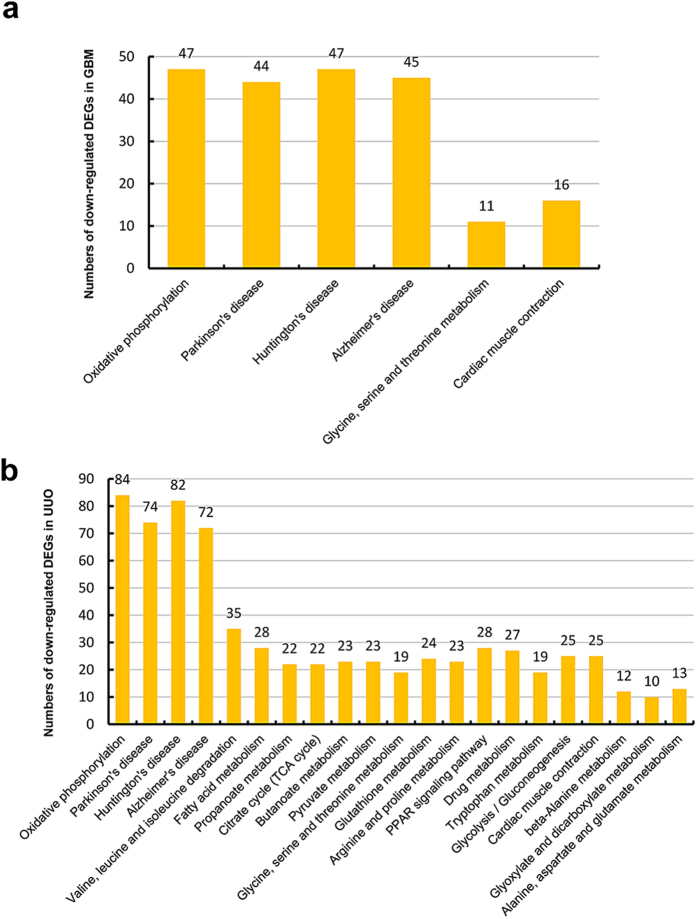
Histogram showing all the significant KEGG pathways of down-regulated genes in WT-GBM (a) or WT-UUO models (b) compared to WT-Nor. WT-UUO:UUO model in wild-type mice; WT-GBM:anti-GBM GN model in wild-type mice; WT-Nor:wild-type normal mice.

To identify the relationship between DEGs and to uncover the common pathways in renal injury, the DEGs were analyzed by KEGG Pathway. The number of significant pathway was shown in Table 5 and all the KEGG analysis in two models was listed in Supplementary File S4 and S5 (including all the pathway terms and gene ID), In WT-GBM and WT-UUO, the significant KEGG pathways based on DEGs were shown in Figs 4 and 5. For up-regulated genes in two models showed in Fig. 4, the common DEGs in WT-GBM and WT-UUO models were enriched in 13 pathways, such as cell adhesion molecules; cytokine-cytokine receptor interaction; hematopoietic cell lineage; chemokine signaling pathway; complement and coagulation cascades; B cell receptor signaling pathway *etc*. For down-regulated genes, 6 common pathways in two WT models were: oxidative phosphorylation; Parkinson’s disease; Huntington’s disease; Alzheimer’s disease; glycine, serine and threonine metabolism; cardiac muscle contraction (Fig. 5).

As same as the results of GO analysis, only a few significant KEGG pathways based on DEGs were found in KO-GBM and KO-UUO models compared to KO-Nor. As listed in Supplementary File S4 and S5, only one pathway (Complement and coagulation cascades) in KO-GBM, 4 pathways (ECM-receptor interaction, Focal adhesion, Cytokine-cytokine receptor interaction, Chemokine signaling pathway) in KO-UUO were found significantly in up-regulated genes related to renal injury. For down-regulated genes, 8 pathways (Oxidative phosphorylation, Parkinson’s disease, Alzheimer’s disease, Huntington’s disease, *etc*) were found in KO-UUO models, but no significant pathway was found in KO-GBM models. None of the KEGG pathway was found regulated by Smad3, which may be largely due to less DEGs were found in KO models.

### Dysregulation of splicing in CKD mouse transcriptome

**Figure 6:**
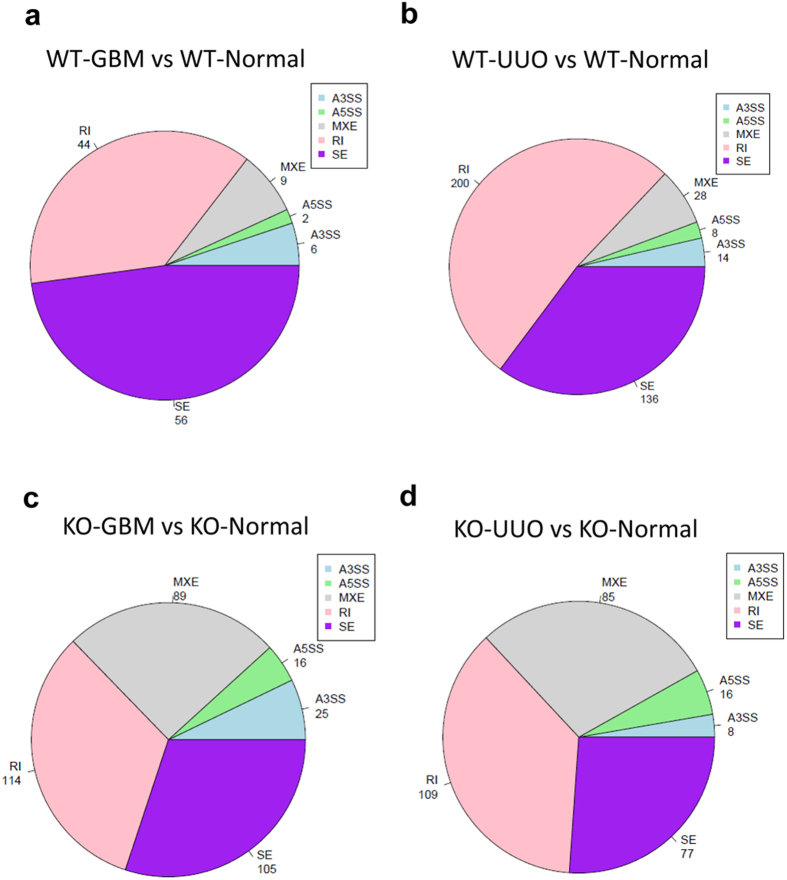
Pie charts showing the number ofthe significant AS genes and events in WT and Smad3 knockout groups. (**a**) WT-GBM compared toWT-Nor. (**b**) WT-UUOcompared to WT-Nor. (**c**) KO-GBM compared toKO-Nor. (**d**) KO-UUO compared to KO-Nor. WT-GBM:anti-GBM GN model in wild-type mice; WT-Nor:wild-type normal mice; WT-UUO:UUO model in wild-type mice; KO-GBM:anti-GBM GN model in Smad3 knockout mice; KO-UUO:UUO model in Smad3 knockout mice; KO-Nor:Smad3 knockout normal mice; SE:SkippingExon; MEX: Mutually Exclusive Exons; A5SS: Alternative 5′ splice site; A3SS: Alternative 3′ splice site; RI: Retained Intron

Alternative splicing (AS) is a mechanism, by which more than one mRNA transcripts are generated from the same mRNA precursor due to variations in the incorporation of coding regions, giving rise to functionally different proteins. We used our RNA-Seq data to detect AS events and genes with alterations in AS between samples genome-widely. Five type of AS models were detected including skipping exon (SE), retention intron (RI), alternative 5′ splice site (A5SS), alternative 3′ splice site (A3SS), mutually exclusive exons (MEX) in our results. All the significant AS genes and events in WT and Smad3 knockout groups were listed in Supplementary File S6. The distribution of AS in disease models compared to normal groups was shown in Fig. 6, the number of significant AS events in WT and Smad3-KO model were listed respectively. SE, and RI are the most prevalent type of AS events in all groups, accounting for about 70% of all detected AS events. It is interesting that the distribution of AS events were changed in the Smad3-KO groups, characterized by the increasing gene number of MXE, compared to WT. In order to identify the functional role and relationship between AS genes, these genes were further analyzed by DAVID GO (Supplementary File S7). and KEGG pathway (Supplementary File S8). Two GO terms of CC (mitochondrial part and mitochondrion) were significant in WT-UUO models, but none in WT-GBM was found. One GO terms of BP (transmembrane transport) and 2 GO terms of MF (coenzyme binding and cofactor binding) were significant in KO-GBM models, while 6 MF (nucleotide binding, adenyl nucleotide binding, *etc*) in KO-UUO models. No significant KEGG pathway was found in all the groups.

### Real-time Quantitative PCR and Western blot Validation

**Figure 7:**
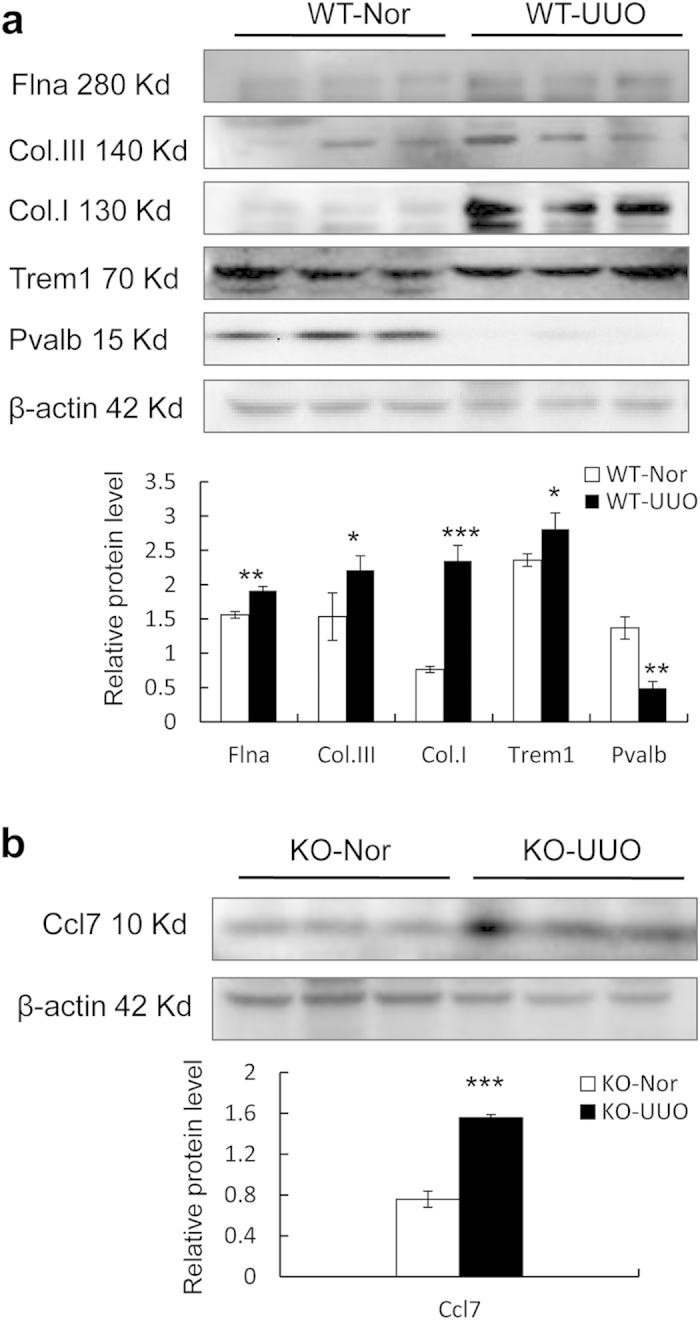
(a) Western blot analysis show protein expression levels of filamin alpha (Flna), collagen III (Col. III), collagen I (Col. I), Trem1, Pvalb. (**b**) Ccl7 expression by western blot analysis. Each bar represents the mean ± SD for at least 6 animals. *p < 0.05, **p < 0.01, ***p < 0.001 vs WT-Nor or KO-Nor.WT-Nor:wild-type normal mice; WT-UUO:UUO model in wild-type mice; KO-Nor:Smad3 knockout normal mice; KO-UUO:UUO model in Smad3 knockout mice.

Validation of DEGs in different models was performed by using real-time qPCR. As shown in Table 6, two groups of DEGs were included: DEGs in wild type mice of UUO and anti-GBM GN models; DEGs in Smad3 knockout mice of UUO and GBM models. Two of most up- and down-regulated genes in each group were selected for validation. Although mRNA variation in fold changes was different between qPCR and RNA-seq, the expression patterns were coincident between the results of these two techniques. Some of the DEGs were also selected for validation at protein level by Western blot. As shown in Fig. 7, the protein level of Flna and Trem1 were also significantly up-regulated in WT-UUO compared to WT-Nor, while Pvalb was down-regulated. We also detect some classical markers in UUO models for justification, the extracelluar matrix protein Collagen type III (Col.III) and Collagen type I (Col.I) were upregulated as our previous reports.

## Discussion

Most CKD patients regardless the initial causes undergo a final common pathway to interstitial fibrosis and glomerulosclerosis. Immunologically-mediated anti-GBM GN and non-immunologically-associated UUO models are two classic models closely related to patients with CKD and were commonly used to investigate the mechanism of CKD. In anti-GBM GN model, a severe and rapidly progressive crescentic GN was developed, including the presence of immunoglobulin complexes and immune cells in the kidney which may cause irreversible chronic renal injury^18^. UUO model generates progressive obstructive nephropathy marked by renal interstitial fibrosis and also hemodynamic and metabolic changes^19^. Although these two typical CKD models are quite different of phenotypic symptoms, they also have some common mechanisms, such as the infiltration of immune cells and excess deposition of the extracellular matrix in the diseased kidney.

We have previously found that TGF-β/Smad3 is essential in the process of CKD, especially in renal fibrosis^5^. Therefore, addressing the pathogenesis of the renal injury in terms of Smad3-targeted molecules will help to develop a specific therapy for CKD. In our previous study, we found that transcripts including microRNAs and long non-coding RNAs are participated in the gene regulation during the CKD development^11^,^12^,^20^,^21^,^22^,^23^. Recently, a research group using deep sequencing to obtain the gene expression profiles of 14 microdissected renal tubule segments from the proximal tubule through the inner medullary collecting duct of normal rat kidneys^24^. Different from their study, the present study, functional annotation-based analysis of DEGs (differentially expressed genes) in CKD revealed two dominant themes: 1) common dysregulation of genes associated with renal injury in two CKD models; and 2) genes associated with Smad3 in renal injury. RNA-Seq was used for screening the common DEGs between two CKD models, and bioinformatics was used to analyze the expression variations, gene function and alternative splicing of DEGs, in order to further identify Smad3-related genes and biological pathways that regulate the pathogenesis of chronic renal injury.

In our study, to identify the signaling molecules and their putative functions in the process of CKD, the mouse kidney tissues of anti-GBM GN at day 10 and UUO at day 5 were selected for RNA-sequencing, which are relative earlier time points in the period of chronic kidney injury, so as to understand the biological changes happened during the pathogenesis and progression of CKD. In the kidney tissue of anti-GBM GN at day 10, a large number of genes related to immune response were significantly increased, while transcripts of surfactant associated protein, channel protein and metabolic enzymes were decreased. Among them, 155 up-regulated genes could be successfully clustered into GO terms of immune response, 101 down-regulated genes ranked into oxidation reduction. In comparison of WT-GBM and WT-Nor, immunoglobulin genes are mostly up-regulated, such as Ighg1, Ighg2c, Igkv, which are closely related to the antibody production of B cell, and have the potential to promote cell growth and proliferation^25^,^26^.

It was previously reported that a number of genes related to immune response and fibrosis were increased on day 7 in anti-GBM GN model, and transcripts of growth factors, channel protein and metabolic enzymes were decreased on around day 11 to 16^27^. It is consistent with our results, supporting the reliability of RNA-sequencing. For further understanding of the biological functions and interactions of these genes, pathway-based analysis was conducted based on the KEGG Pathway database. Results of KEGG pathway were similar with the GO analysis, the significant enriched pathways for up-regulated genes in WT-GBM were cell adhesion molecules, cytokine receptor interaction, ECM-receptor interaction, while down-regulated DEGs were enriched in the pathways of oxidative phosphorylation, Parkinson’s disease, Huntington’s disease, Alzheimer’s disease.

In the UUO model, the mostly-induced genes in WT-UUO were coding for cytoskeleton proteins, such as FlnA (filamin, alpha) and moesin, which are important to the remodeling of cytoskeleton and modulation of cell shape and migration^28^. In addition, a number of inflammatory and immune response genes are also up-regulated in UUO. This speculation consistent with the GO analysis, up-regulated genes at the UUO day 5 could be successfully clustered into GO terms of immune response, response to wounding, inflammatory response, positive regulation of immune system process, defense response, and cell adhesion. For down-regulated genes, they were enriched in GO terms of oxidation reduction, generation of precursor metabolites and energy, electron transport chain, cofactor metabolic process, coenzyme metabolic process, fatty acid metabolic process, transmembrane transport. According to the pathway analysis of WT-UUO, by comparing to WT-Nor, genes associated with cytokine-cytokine receptor interaction, chemokine signaling pathway, focal adhesion, ECM-receptor interaction were significantly upreguated, whereas, those related to the pathway of oxidative phosphorylation Parkinson’s disease, Huntington’s disease, Alzheimer’s disease were downregulated.

As expected, alterations of DEGs found in these two models exhibited common mechanisms in the development of renal injury. In both anti-GBM GN and UUO models, the functions of commonly up-regulated genes were associated with cell adhesion, inflammation and immune response at the early stage of CKD. KEGG pathway network analysis showed that genes belong to cell adhesion molecules, cytokine-cytokine receptor interaction, hematopoietic cell lineage, chemokine signaling pathway, complement and coagulation cascades, B cell receptor signaling pathway were commonly up-regulated. In contrast, the commonly down-regulated genes are related to material or electron transportation and metabolism, their KEGG pathway were oxidative phosphorylation, Parkinson’s disease, Huntington’s disease, Alzheimer’s disease, glycine, serine and threonine metabolism, cardiac muscle contraction. Although, Parkinson’s disease (PD), Huntington’s disease (HD), Alzheimer’s disease (AD) are neurodegenerative disorders cause serious movement or cognitive disability, the proteins in these pathways also involved in multiple cell functions and DEGs enriched in these pathways are potentially participated in renal disease. In PD pathway, mutations or altered expression of proteins in this pathway contributes to the damage and subsequent loss of dopaminergic neurons through common mechanisms including proteasome dysfunction, mitochondrial impairment, and oxidative stress^29^,^30^. The mutation of proteins in HD pathway also may lead to abnormal endocytosis, augmented p53 mediates mitochondrial dysfunction, as well as Ca^2+^ signaling disorder^31^,^32^,^33^. AD associated proteins have various pathological effects on cell and organelle function through activation of cell surface death receptors, disrupting mitochondria function, and triggering calcium dysfunction^34^,^35^.

These results imply that progressive renal fibrosis may begin with inflammatory response after injury, whereas, remodeling of the cytoskeleton in renal cells was the initial stage of fibrosis. At this stage, the inflammatory and immune system is activated, accompanied with the cytoskeleton reconstruction and the extracellular matrix deposition. At the same time, the normal cell functions, such as metabolism, oxidative phosphorylation, material transportation and some signal transduction were dramatically suppressed.

To further investigate the pathogenic role of Smad3 at the early stage of chronic renal injury, two CKD models were performed on Smad3 knockout mice. Our results revealed that, compared to WT mice, deletion of Smad3 largely altered the gene expression profiles of these two diseases. It is consistent with our previous observations that fibrogenesis was prevented in Smad3 KO UUO mice and a loss of Treg and enhanced Th-2 and Th-17-mediated renal injury in anti-GBM GN mice lacking Smad3^36^. In anti-GBM GN and UUO models, the number of DEGs related to Smad3 was 55 and 39 respectively. Most of the Smad3-related DEGs in anti-GBM model have putative functions in immune response, since 46 immunoglobulin genes were found in the total of 55 DEGs. In addition, Smad3-related DEGs in UUO model were belonged to immune response, but also exhibited distinct biological functions that were enriched in organic anion transporter and metabolism. Only 9 common DEGs were related to Smad3 in these two models, including 6 immunoglobulin genes (Ighg1, Ighg2c, Igkv12-41, Ighv14-3, Ighv5-6 and Ighg2b) and 3 metabolic genes (Ugt2b37, Slc22a19, and Mfsd2a). Ugt2b37 (UDP-glucuronosyltransferases) is a metabolic enzymes involved in the metabolism of key endogenous compounds including bilirubin, bile acids, fatty acids, steroid hormones, thyroid hormones and fat soluble vitamins^37^. Slc22a19 belongs to organic anion transporter family, also named organic anion transporter 5 (Oat5), is a renal organic anion/dicarboxylates exchanger under physiological conditions^38^,^39^. Mfsd2a (major facilitator super family domain containing 2a), usually considers as the major transporter for docosahexaenoic acid (DHA) and a key regulator of ‘blood-brain barrier'^40^,^41^. but also found functions in modulating cell cycle and matrix attachment by inducing a G1 block, and then suppress tumor cells by impairing adhesion and migration^42^. These common DEGs related to Smad3 are all have potential roles in renal injury.

Alternative splicing (AS) is a regulated mechanism during gene expression, during this process, particular exons of a gene may be included within or excluded from the final processed mRNA produced from that gene. Since Smad3 can alter the gene expression profile, we also use our RNA-Seq data to detect AS events and genes with alterations in AS between CKD and normal groups. The results shown that the distribution of AS events were changed in the Smad3-KO groups, characterized by the increasing gene number of MXE, indicated that deficiency of Smad3 may have impact on gene splicing.

Regrettably, no significance common pathway and very rare significant GO term were found in the CKD models lacking of Smad3. This may largely be due to limited DEGs that were found in the Smad3 KO models. By the RNA-seq based transcriptome analysis, we found the central role for Smad3 in the immune response of B cell, which can regulate the transcription of immunoglobulin. The results could explain the phenomenon that blocks renal fibrosis by targeting Smad3 may cause autoimmune diseases^5^,^43^.

To summarize, our transcriptome analysis of two mice CKD models suggested that increased expression of inflammation and immune response related genes, and cytoskeletons may be as biological markers at the early stage of chronic renal injury. Furthermore, we demonstrated that Smad3 is a critical transcription factor in the immune response, which can regulate the transcription of immunoglobulins. It implies the important role of Smad3 in the immune-mediated glomerulonephritis. Besides, there are some limitations to this study since the UUO and anti-GBM GN models may not be able to completely recapitulate the human CKD phenotype. The sample size used to perform RNA-sequencing is too small, and the usage of whole kidney tissue for RNA-sequencing reduces sensitivity for detecting cell type specific changes of transcripts, particularly in those with low abundance. Nevertheless, our findings from this study identify the downstream signaling molecules of Smad3 may have potential functions in renal injury; it may provide precise and potential therapeutic targets for CKD.

## Methods

### Mouse Model of UUO and Anti-GBM GN

The CKD models of UUO and anti-GBMGN were induced in both sexes of C57BL/6J wild-type (WT) and Smad3 knockout (KO) mice at 8 to 10 weeks of age, as previously described^3^,^44^. Briefly, for the UUO model, the left ureter from Smad3 WT or KO mice (n = 8 per group, both sexes, 8weeks of age, 22 to 25 g body weight) was ligated and the UUO kidney was harvested at day 5 after surgery for further analysis. For the anti-GBMGN model, Smad3 WT or KO mice were preimmunized with sheep IgG in Freund’s complete adjuvant(Sigma Chemical Co, St. Louis, MO), followed 5 days later by i.v. administration of sheep anti-mouse GBM immunoglobulin at a dose of 60 mg/g of body weight (termed day 0). Diseased mice were euthanized at day 10 for further analysis. Groups of age- and weight-matched normal Smad3 WT or KO mice were used as normal controls. The experimental procedures for both models were performed following the approved protocol by the Animal Experimentation Ethics Committee at The Chinese University of Hong Kong and the experimental methods were carried out in accordance with the approved guidelines.

### Sample collection and RNA isolation

Kidney tissues of Smad3 KO/WT were collected from normal, UUO model at day 5, and anti-GBM GN model at day 10 for RNA sequencing (n = 2 in each group). Total RNA from kidneys was isolated by using Trizol reagent (Life Technologies, Carlsbad, CA, USA) in accordance with the manufacturer’s instructions, and then followed by an additional DNase I digestion to remove genomic DNA contamination. RNA quality and purity was checked by using the ND-1000 Nanodrop. And then total RNA samples were used to remove highly abundant ribosomal RNAs before sequencing.

### RNA sequencing and reads quantifying, mapping, assembling

The sequencing was done by Illumina Genome Analyzer (serial number Hiseq2000) as previously reported^11^,^12^. Both library building and sequencing were performed by Beijing Genomics Institute (BGI), Shenzhen, China. Low-quality reads, over 50% of the sequences reads (including adapters) has quality score ≤10 and N > 5%, were discarded. Ribosome RNA sequences were filtered from the raw fragments. Those left clean reads were mapped to genome (UCSC genome browser, Version: July.2007, mm9) by using Tophat[1.3.0.Linux_x86_64 (bowtie-0.12.7)] and then assembled with Cufflinks (1.3.0.Linux_x86_64)^45^. The sequencing data obtained were available at the Gene Expression Omnibus Web site (http://www.ncbi.nlm.nih.gov/geo/) under accession GSE55808. All the analysis results of gene expression are also provided as a webpage (http://songyanglab.sysu.edu.cn/Transcriptome_analysis_in_renal_injury).

### Analysis of differentially expressed genes

The FPKM (expected number of fragments per kilobase of transcript sequence per millions base pairs sequenced) were used to Quantifying the mRNA expression, which measure of read density reflects the molar concentration of a transcript in the starting sample by normalizing for RNA length and for the total read number in the measurement^46^. The genes with false discovery rate (FDR = adjusted p-values) <0.05 and fold change (FC)>2 were considered to indicate significant up-regulation; FDR<0.05 and FC<0.5 were considered as significant down-regulation; FC between 0.5 and 2 were considered as no significance. The DEGs were analyzed by software edgeR (Release 3.1)^47^. All statistical analyses were performed in R. For the identification of Smad3-related genes, DEG has different expression direction in WT and Smad3 KO mice will be considered as Smad3-related, such as genes up-regulated in WT but suppressed in Smad3 KO, or down-regulated in WT but up-regulated in Smad3 KO. Pearson’s correlation coefficient based on transcript abundance(FPKM)were used to measure the gene expression similarity between samples.

### Gene Ontology and Pathway Analysis

Significantly up and down-regulated genes were analyzed for enrichment of gene ontology (GO) terms (biological processes, molecular function and cellular component) and KEGG Pathway by using DAVID bioinformatics resources^48^. For GO term and Pathway analysis, Probabilities were evaluated by Bonferroni correction and FDR values less than 0.05 were considered as significant.

### Detection and quantification of alternative splicing variants

The Multivariate Analysis of Transcript Splicing (MATS,Release 2.0.0) was used to detect differently alternative splicing events across samples^49^. The Ensembl annotation was supplied to STAR to ensure the sensitivity of aligning spliced fragments to known junctions^50^. Generally, the alternative splicing events were classified into seven different types according to the structures of exons. These seven types include skipping exon (SE), retention intron (RI), alternative 5′ splice site (A5SS), alternative 3′ splice site (A3SS), mutually exclusive exons (MEX), alternative first exons (AFE), and alternative last exons (ALE) as described by Wang *et al*.^51^,^52^. Because of the unperfected algorithms for AFE and ALE, only the remaining five alternative splicing models listed above were analyzed and presented in this study. We detected the differently alternative splicing events by using the cuff-off criteria as FDR <0.1 to filter the result of MATS. The genes has differential expression of splicing variants were used for gene Ontology and pathway analysis as described above.

### Quantitative real-time PCR (qPCR)

Gene expression was quantified by real-time PCR with SYBR green Permix Kit(Life Technologies, Carlsbad, CA). Quantitative real-time PCR was carried out on ABI7900 system using the following program: 50 °C for 2 min 95 °C for 10 min; 40 cycles of 95 °C for 15 sec, 58 °C for 15 sec, and 72 °C for 30 sec, and then dissociation steps. Primers for all the tested DEGs and β-actin, an endogenous control for normalization, are listed in Supplementary Table 1. The relative levels of target genes were calculated by using 2^−ΔΔCt^ method^53^. Statistical analyses were performed using one-way ANOVA followed by Newman-Keuls multiple comparison test from GraphPad Prism 5.0 (Graph Pad Software, San Diego, CA).

### Western blot

Protein from kidney tissues was extracted by using the protein lysis buffer(Cell Signaling,Danvers, MA, USA) and then quantified by the Bradford assay (Bio-Rad, USA) . An equal amount of protein was separated on SDS-polyacrylamide gels and transferred onto nitrocellulose membranes (Amersham Biosciences, USA). After blocking non-specific binding with 5% skim milk in TBST for 1 h at room temperature, membranes were then incubated overnight at 4 °C with the primary antibody against Flna (Cell Signaling, Danvers, MA, USA), Col. III and Col. I (Southernbiotech, USA), Trem1 and Pvalb (boster,Wuhan, China), β-actin(Santa Cruz, USA), followed by horseradish peroxidase-conjugated secondary antibody for 1 h at room temperature. The ratio for the protein examined was normalized against β-actin and was expressed as the mean ± SD.

## Additional Information

**How to cite this article**: Zhou, Q. *et al*. Identification of Genes Associated with Smad3-dependent Renal Injury by RNA-seq-based Transcriptome Analysis. *Sci. Rep*. **5**, 17901; doi: 10.1038/srep17901 (2015).

## Supplementary Material

Supplementary Information

Supplementary File S1

Supplementary File S2

Supplementary File S3

Supplementary File S4

Supplementary File S5

Supplementary File S6

Supplementary File S7

Supplementary File S8

## Figures and Tables

**Table 1:** Mapping overview of RNA-sequencing reads on the mouse genome.

Sample	All reads	All reads (pairs)	Mapped reads (all)	Mapping rate	Unique mapped	Unique mapped rate
WT-Nor-1	51398366	25699183	25006455	97.30%	21883540	85.15%
WT-Nor-2	52300544	26150272	25528188	97.62%	22918918	87.64%
WT-UUO-1	51708360	25854180	18332567	70.91%	13718644	53.06%
WT-UUO-2	51913666	25956833	24953074	96.13%	22352312	86.11%
WT-GBM-1	52125094	26062547	24740414	94.93%	21836781	83.79%
WT-GBM-2	54110904	27055452	26238539	96.98%	22334706	82.55%
KO-Nor-1	53474448	26737224	25800912	96.50%	22272463	83.30%
KO-Nor-2	53680294	26840147	25895801	96.48%	21477366	80.02%
KO-UUO-1	54354462	27177231	22326568	82.15%	15611253	57.44%
KO-UUO-2	51186728	25593364	24937874	97.44%	22492658	87.88%
KO-GBM-1	54445046	27222523	18228106	66.96%	11609579	42.65%
KO-GBM-2	51855890	25927945	24349590	93.91%	20020045	77.21%

Note: WT-Nor:wild-type normal mice; WT-UUO:UUO model in wild-type mice; WT-GBM:anti-GBM GN model in wild-type mice; KO-Nor:Smad3 knockout normal mice; KO-UUO:UUO model in Smad3 knockout mice; KO-GBM: anti-GBM GN model in Smad3 knockout mice.

**Table 2:** The number of differentially expressed genes in two CKD models.

Samples	Total	Up (%)	Down (%)
WT-GBM vs WT-Nor	2417	1552 (64.21%)	865 (35.79%)
WT-UUO vs WT-Nor	4959	2898 (58.44%)	2061 (41.56%)
Common in two WT models	1922	1203 (62.59%)	719 (37.41%)
KO-GBM vs KO-Nor	243	117 (48.15%)	126 (51.85%)
KO-UUO vs KO-Nor	735	400 (54.42%)	335 (45.58)
Common in two KO models	84	36 (42.86%)	48 (57.14%)

Note: WT-GBM:anti-GBM GN model in wild-type mice; WT-Nor:wild-type normal mice; WT-UUO:UUO model in wild-type mice; KO-GBM:anti-GBM GN model in Smad3 knockout mice; KO-UUO:UUO model in Smad3 knockout mice; KO-Nor:Smad3 knockout normal mice; Up:up-regulated genes; Down:down-regulated genes.

**Table 3:** The number of differentially expressed genes in renal injury related to Smad3.

Comparision	GBM related to Smad3	UUO related to Smad3	Common	Gene name
Up in WT & Down in KO	50	17	6	Ighg1, Ighg2c, Igkv12-41, Ighv14-3, Ighv5-6, Ighg2b
Down in WT & Up in KO	5	22	3	Ugt2b37, Slc22a19, Mfsd2a

Note: WT:anti-GBM GN or UUO model in wild-type mice; KO:anti-GBM GN or UUO model in Smad3 knockout mice; Up:up-regulated genes; Down:down-regulated genes.

**Table 4:** The number of significant GO of differentially expressed genes.

Regulated direction	GO	WT-GBM vs WT-Nor	WT-UUO vs WT-Nor	Common in WT	KO-GBM vs KO-Nor	KO-UUO vs KO-Nor	Common in KO
UP-regulation	BP	171	209	138	0	22	0
	MF	9	34	9	0	13	0
	CC	12	23	10	1	9	1
Down-regulation	BP	26	105	26	5	14	1
	MF	16	52	16	5	25	4
	CC	16	28	16	4	13	1

Note:WT-GBM:anti-GBM GN model in wild-type mice; WT-Nor:wild-type normal mice; WT-UUO:UUO model in wild-type mice; KO-GBM:anti-GBM GN model in Smad3 knockout mice; KO-UUO:UUO model in Smad3 knockout mice; KO-N-or:Smad3 knockout normal mice; BP:biological processes; MF:molecular function; CC:cellular component.

**Table 5:** The number of significant KEGG pathway of differentially expressed genes.

Regulated direction	WT-GBM vs WT-Nor	WT-UUO vs WT-Nor	Common in WT	KO-GBM vs KO-Nor	KO-UUO vs KO-Nor	Common in KO
UP-regulation	17	19	13	1	4	0
Down-regulation	6	21	6	0	8	0

Note: WT-GBM:anti-GBM GN model in wild-type mice; WT-Nor:wild-type normal mice; WT-UUO:UUO model in wild-type mice; KO-GBM:anti-GBM GN model in Smad3 knockout mice; KO-UUO:UUO model in Smad3 knockout mice; KO-Nor:Smad3 knockout normal mice.

**Table 6:** Validation of RNA-sequencing results by real-time PCR.

Comparison	Gene	Regulated direction	FC by qRT-PCR	p-value by qRT-PCR	LogFC by RNA-seq	FDR by RNA-seq
WT-GBM vs WT-Nor	Ighg1	Up	161.1160	0.0640	13.2864	6.93E–30
	Igkc	Up	2.2981	0.0020	9.9349	4.04E–32
	Cox6a2	Down	0.3434	0.0456	−5.6157	5.25E–10
	Angptl7	Down	0.2022	0.0043	−5.6949	4.17E–46
	Sftpc	Down	0.0235	0.0246	−11.4023	5.63E–05
WT-UUO vs WT-Nor	Il1rn	Up	105.1993	0.0006	10.5668	7.98E–26
	Trem1	Up	4.0638	0.0185	8.7348	1.19E–18
	Flna	up	6.2702	0.0070	17.2533	1.40E–54
	Pvalb	Down	0.0051	0.0000	−6.9307	2.20E–13
	Ugt1a9	Down	0.4222	0.0001	−6.2263	1.81E–11
	Sftpc	Down	0.0591	0.0270	−11.3729	8.30E–05
KO-GBM vs KO-Nor	Rpl29	Up	1.8738	0.0331	7.0270	9.69E–16
	Ceacam2	Up	6.3943	0.0179	5.4004	1.91E–13
	Igj	Down	0.1877	0.0023	−3.5747	2.70E–05
	Hrg	Down	0.3559	0.0379	−8.21951	4.07E–04
KO-UUO vs KO-Nor	Col11a1	Up	5.5698	0.0071	6.9993	5.48E–07
	Ccl7	Up	23.4986	0.0055	7.228708	4.57E–02
	Cyp3a11	Down	0.4963	0.0165	−7.8656	5.78E–08
	Serpina1a	Down	0.7025	0.0470	−8.8200	2.54E–20

Note: WT-GBM:anti-GBM GN model in wild-type mice; WT-Nor:wild-type normal mice; WT-UUO:UUO model in wild-type mice; KO-GBM:anti-GBM GN model in Smad3 knockout mice; KO-UUO:UUO model in Smad3 knockout mice; KO-Nor:Smad3 knockout normal mice.
