# Supplementary material for: Identification of Genes Associated with Smad3-dependent Renal Injury by RNA-seq-based Transcriptome Analysis
Source: Sci Rep. 2015 Dec 9;5:17901. doi: 10.1038/srep17901 (PMC4673424; doi:10.1038/srep17901)
Supplement: Supplementary Information [file srep17901-s1.pdf]

Supplementary Information

Identification of Genes Associated with Smad3-dependent Renal Injury by RNA-seq-based Transcriptome Analysis

Qin Zhou[1,2,+], Yuanyan Xiong[4,5,+], Xiao R Huang[2,3], Patrick Tang[2], Xueqing Yu[1,*], Hui Y. Lan[2, 3,*]

[1]Department of Nephrology, The First Affiliated Hospital, Sun Yat-sen University, Guangzhou, China; [2]Li Ka Shing Institute of Health Sciences and Department of Medicine & Therapeutics, the Chinese University of Hong Kong, Hong Kong, China; [3]Shenzhen Research Institute, the Chinese University of Hong Kong, Shenzhen, China. [4]State Key Laboratory for Biocontrol, Sun Yat-sen University, Guangzhou, China. [5]SYSU-CMU Shunde International Joint Research Institute, Guangzhou, China.

[+]These authors contributed equally to this work

*Corresponding authors:

Dr. Hui Y. Lan, Li Ka Shing Institute of Health Sciences, and Department of Medicine and Therapeutics, The Chinese University of Hong Kong, Hong Kong, China, Tel: 852-3763 6077, Fax: 852-2145 7190, Email: hylan@cuhk.edu.hk; and Dr. Xueqing Yu, Department of Nephrology, The First Affiliated Hospital, Sun Yat-sen University, 58th, Zhongshan Road II, Guangzhou, China, Tel: 86-20-87766335, Fax 86-20-87769673, E-mail: yuxq@mail.sysu.edu.cn

Figure S1. **Pearson correlation of FPKM values between groups.** WT.GBM: anti-GBM GN model in wild-type mice; WT normal, wild-type normal mice; WT UUO, UUO model in wild-type mice; Smad3 GBM, anti-GBM GN model in Smad3 knockout mice; Smad3UUO, UUO model in Smad3 knockout mice; Smad3Nor, Smad3 knockout normal mice. Correlation coefficient value *>0.6, **>0.8, ***>0.9.

Table S1. Real-Time PCR Primers for differentially expressed genes.

File S1.All DEG comparison in wild-type and Smad3 knockout groups. (see separate Excel documents)

File S2. DAVID GO analysis of up-regulated genes in wild-type and Smad3 knockout groups. (see separate Excel documents)

File S3. DAVID GO analysis of down-regulated genes in wild-type and Smad3 knockout groups. (see separate Excel documents)

File S4. KEGG Pathway analysis of up-regulated genes in wild-type and Smad3 knockout groups. (see separate Excel documents)

File S5. KEGG Pathway analysis of down-regulated genes in wild-type and Smad3 knockout groups. (see separate Excel documents)

File S6. Different Alternative splicing genes and events in wild-type and Smad3 knockout groups (see separate Excel documents)

File S7. DAVID GO analysis of Different Alternative splicing genes (see separate Excel documents)

File S8. KEGG pathway analysis of Different Alternative splicing genes (see separate Excel documents)

Supplementary Figure 1

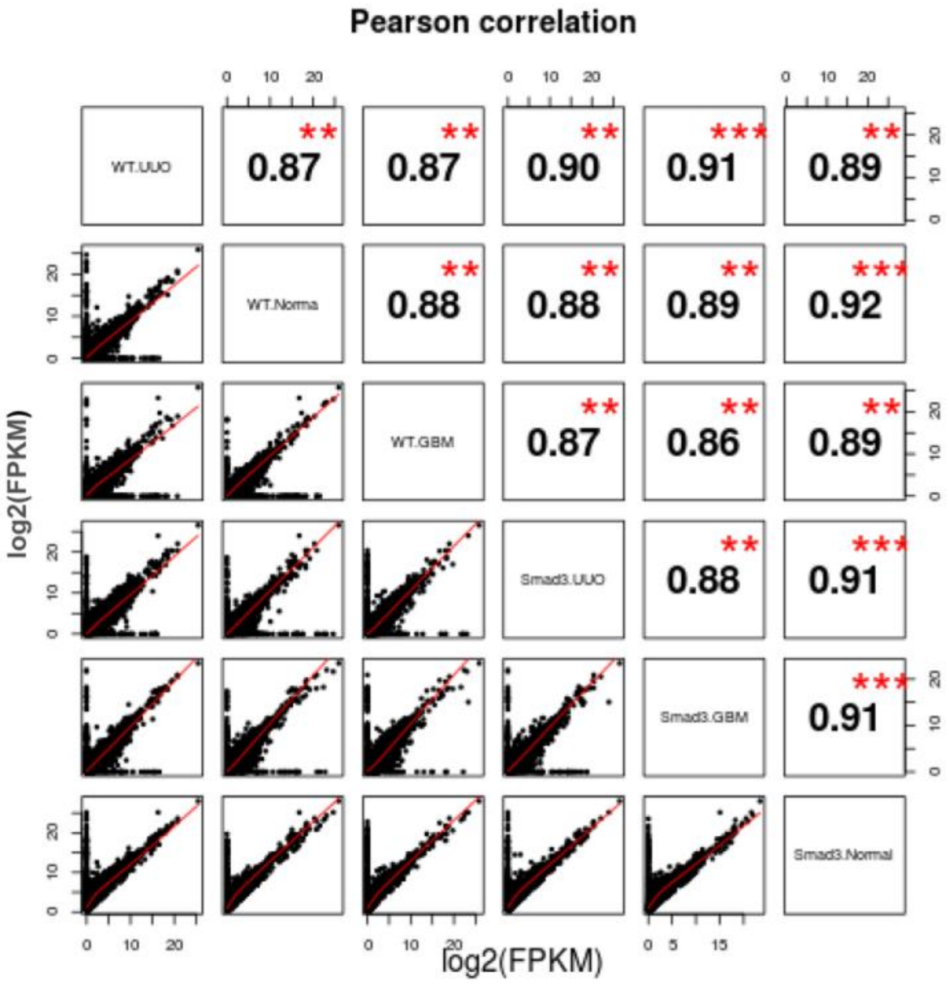

Supplementary Table 1. Real-Time PCR Primers for differentially expressed genes.

Gene	Forward	Reverse
**Ighg1**	GCACACAGCTCAGACGAAACC	TCCTTGCCATTGAGCCAGTCC
**Igkc**	ATGCTGCACCAACTGTATCCA	CCAACTGTTCAGGACGCCAT
**Cox6a2**	CTGCTCCCTTAACTGCTGGAT	GATTGTGGAAAAGCGTGTGGT
**Angptl7**	TGACTGTTCTTCCCTGTACCA	CAAGGCCACTCTTACGTCTCT
**Sftpc**	TCCTCGTTGTCGTGGTGATTG	GGAAAAGGTAGCGATGGTGTC
**Il1rn**	GCTCATTGCTGGGTACTTACAA	CCAGACTTGGCACAAGACAGG
**Trem1**	GACTGCTGTGCGTGTTCTTTG	GCCAAGCCTTCTGGCTGTT
**Flna**	GGCTACGGTGGGCTTAGTC	GTGGGACAGTAGGTGACCCT
**Pvalb**	ATCAAGAAGGCGATAGGAGCC	GGCCAGAAGCGTCTTTGTT
**Ugt1a9**	TCTCGCTCCCATCAGTAATCTT	TGGTTCCACACTCTCTCCTTG
**Rpl29**	CAAGTCCAAGAACCACACCAC	GCAAAGCGCATGTTCCTCAG
**Ceacam2**	TGGCAGAGAGGCACTATACAG	TGGAGTTGTTGCTTGTGATGT
**Igj**	TGACGACGAAGCGACCATTC	TTCAAAGGGACAACAATTCGGA
**Col11a1**	ACAAAACCCCTCGATAGAAGTGA	CTCAGGTGCATACTCATCAATGT
**Cyp3a11**	GCCACTCACCCTGATATCCA	CTGAGGGTTTCATTAAGCACCA
**Serpina1a**	CCAGCCTCCCATGAGATCGCTA	GAGTGTCACCCTTGCTCCC
**β-actin**	CCCTGAAGTACCCCATCGAGCACG	GGTCATCTTCTCGCGGTTGGCCT
